# Evaluating a Train-the-Trainer program to implement a navigation program for older people with cancer across six European countries, as part of the EU NAVIGATE project: a Kirkpatrick multi-method evaluation

**DOI:** 10.1186/s12913-026-14717-6

**Published:** 2026-05-21

**Authors:** Fien Van Campe, Tinne Smets, Iris Beijer Veenman, Lara Pivodic, Barb Pesut, Else Gien Statema, Wendy Duggleby, Nele Van Den Noortgate, Katarzyna Szczerbińska, Barbara Gomes, Andrew Davies, Davide Ferraris, Bregje Onwuteaka-Philipsen, Sara Alfieri, Lieve Van den Block, Kenneth Chambaere

**Affiliations:** 1 Department of Family Medicine and Chronic Care, Vrije Universiteit Brussel, Brussels, Belgium; 2 Department of Public Health and Primary Care, Universiteit Gent, Ghent, Belgium; 3 Department of Public and Occupational Health, Amsterdam UMC, Vrije Universiteit Amsterdam, Expertise Center for Palliative Care Amsterdam UMC, Amsterdam, Netherlands; 4 University of British Columbia Okanagan, Kelowna, BC Canada; 5 University of Alberta, Edmonton, AB Canada; 6 Laboratory for Research on Aging Society, Epidemiology and Preventive Medicine, Medical Faculty, Jagiellonian University Medical College, Kraków, Poland; 7 Faculty of Medicine, University of Coimbra, Coimbra, Portugal; 8 Cicely Saunders Institute of Palliative Care, Policy & Rehabilitation, King’s College London, London, UK; 9 Trinity College Dublin, Dublin, Ireland; 10 Lega Italiana per la lotta contro i tumori di Milano, LILT Milano Monza Brianza, Milano, Italy; 11 Clinical Psychology Unit, Fondazione IRCCS Istituto Nazionale dei Tumori, INT, Milano, Italy; 12 End-of-Life Care Research Group, Universiteit Gent, Corneel Heymanslaan 10, Gent, 9000 Belgium

**Keywords:** Train-the-Trainer program, Patient navigation, Older people, Cancer, Europe, Community support, Public health

## Abstract

**Background:**

Navigation programs support people and families by addressing barriers to care and mitigating physical, psychosocial, and practical challenges. While navigation programs are increasingly recognized within European healthcare systems, evidence on implementation strategies for scale-up across settings such as a Train-the-Trainer program remains limited and rarely comprehensively evaluated. This study evaluates a Train-the-Trainer program developed within the EU NAVIGATE project as an implementation strategy to deliver a navigation program for older people with cancer living at home in six European countries.

**Methods:**

The multi-method evaluation follows the four levels of the Kirkpatrick model. Levels 1 and 2 (Reaction and Learning) were assessed via questionnaires completed by trainers; Level 3 (Behavior) through weekly diaries and group interviews with (international) trainers; and Level 4 (Results) through questionnaires completed by navigators and the intervention beneficiaries (older people with cancer). Quantitative data were analyzed descriptively; qualitative data were analyzed using qualitative content or narrative analysis.

**Results:**

At Levels 1 and 2, trainers (*n* = 11) rated the program as highly useful on a five-point Likert-scale ranging from 1 to 5 (median = 5, IQR = 0.75), reporting strong alignment with their role, clear understanding of the program, and high confidence (median scores 4–5). At Level 3, according to weekly diaries and group interviews, trainers applied the competencies they learned. Successes included positive navigator feedback and training adaptation. Challenges included workload and time constraints, limited team collaboration, and difficulties adapting materials. The ability to contextually adapt the training was facilitated by applying the competencies. At Level 4, reported high levels of overall understanding and confidence following the training, with median scores of 4 (IQR = 1) for both outcomes on a 5-point Likert scale, with highest scores reported for addressing quality of life, and lower scores for supporting technology use. Older people with cancer (*n* = 101) responded that navigators generally demonstrated the intended competencies with median scores ranging from 1 (IQR = 2) to 5 (IQR = 1) on a 5-point Likert scale.

**Conclusion:**

Findings demonstrate the potential of training to implement a navigation program to improve long-term age-appropriate support, emphasize the importance of contextually adapting to local settings, and underline the need for further research to enhance the transferability of navigation programs across diverse health systems.

**Trial registration:**

Clinicaltrials.gov: identifier NCT06110312 (2023/10/31).

**Supplementary Information:**

The online version contains supplementary material available at 10.1186/s12913-026-14717-6.

## Background

Navigation programs aim to support people and their families by addressing barriers to care within health systems, facilitating access to services, and mitigating the challenges posed by illness on physical, psycho-social, and practical levels [1]. A navigator, often a dedicated person, a professional or volunteer with or without a healthcare background, plays a central role in these programs by guiding people through fragmented health and social care systems, advocating for needed services, and/or empowering them to make informed decisions [2]. Navigation programs exist in the entire continuum of care, from diagnosis through treatment, survivorship and end-of-life care [3].

In recent years, there is growing recognition of the potential of navigation programs [4] and increasing interest to embedding such programs into health care policy, accreditation standards, and professional guidelines, mostly in North-America and Europe [5]. Nearly all standards and guidelines indicate that appropriate training for navigators is essential for successful implementation and effectiveness, providing navigators with the tools and competencies necessary to effectively tailor their support to the diverse and complex needs of those they support [6, 7].

A systematic review of research on training in patient navigation programs showed that their evaluation remains limited [8]. When assessments are carried out, they primarily focus on describing the curriculum and the competencies of navigators, the direct recipients of the training, but the full scope of impact of these training programs to implement navigation programs is rarely evaluated from the perspective of other actors involved, such as trainers, navigators, and program beneficiaries.

Various models and approaches have been developed to evaluate training programs. One widely used framework is the four-level Kirkpatrick Model [9]. Level 1 assesses participants’ reactions to the training such as satisfaction; level 2 evaluates the extent of learning such as knowledge and skill acquisition; level 3 examines changes in behavior or application on the job; and level 4 evaluates the results or outcomes, such as program beneficiaries feeling better informed on available social or healthcare services [10]. This comprehensive model has been positively evaluated, in comparison with other evaluation models for training programs, for its clear structure, practicality and approach to evaluate the layered effects of a training program [11, 12].

Within the EU NAVIGATE project [13, 14] we implemented and evaluated a navigation program for older people in declining health with cancer and their families living at home in six European countries: Belgium, Ireland, Italy, the Netherlands, Poland, and Portugal. The program is an adaptation of the Canadian Nav-CARE© (Connecting, Advocating, Resourcing, and Engaging) program [15]. In EU NAVIGATE, trained and mentored volunteers or professional navigators visit or contact older people (aged 70 years or over) with cancer and declining health approximately every two weeks for over a year. These navigators support the program beneficiaries in navigating the health and social care systems and community resources based on their needs and facilitate access to the appropriate services, aiming to address their quality-of-life concerns.

**Fig. 1:**
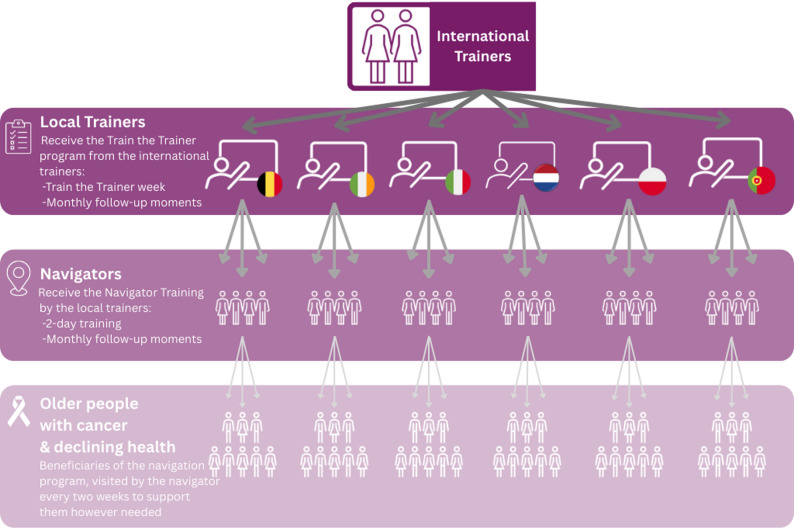
Train-the-Trainer model for the EU NAVIGATE project

A Train-the-Trainer approach was used as an implementation strategy to build local training capacity and ensure program reach across diverse health- and social-care systems [16, 17]. In our Train-the-Trainer model, international trainers train local trainers, who in turn train local navigators to prepare them to help older people with cancer and their families navigate through the health and social care systems (Fig. 1). In this way, the international trainers equip local trainers with the knowledge and skills to master the program content and facilitate trainings. These local trainers often have intimate understanding of contextual issues and more direct knowledge of and access to the settings and communities they are training within, in comparison with international trainers or implementers [18]. Box 1 outlines a description of the navigation program, navigator training, Train-the-Trainer program and adaptation process for the EU NAVIGATE project.

This article aims to evaluate the Train-the-Trainer program used to implement the navigation intervention within the EU NAVIGATE project, based on an in-depth analysis of the four levels of the Kirkpatrick model and from the perspectives of all actors involved: international and local trainers, navigators and older people with cancer and declining health. Table 1 outlines the sub-research questions.

**Box 1:** Description of navigation program, navigator training, Train-the-Trainer program and adaptation process for the EU NAVIGATE project

Navigation program & navigator training
Volunteers or professional navigators are matched with older people with cancer based on mutual interests, and they have contact with each other every two weeks for over a year. Navigators support these older people in navigating the health and social care systems, in order to improve their quality of life and social support. Navigators’ key competencies are addressing quality of life concerns, overcoming barriers to resources and support, facilitating community connections, promoting active engagement, and supporting them in using technology. The Navigator training consists of a two-day training, structured into different modules based on the key competencies for navigators in combination with monthly follow-up meetings of the navigation coordinator with all navigators for ongoing support, education, and experience-sharing among navigators.
**Adaptation process**
The EU NAVIGATE Navigator training is based on the Canadian Nav-CARE© curriculum, which has been rigorously developed, tested and positively evaluated by the Canadian volunteer navigators [19, 20]. Since implementations settings and contexts differ between Canada and Europe, adaptation of the format and content of the training is a recommended implementation strategy to improve the intervention-context fit while maintaining the program’s integrity. Our adaptation process was informed by the ADAPT guidance [21], which emphasizes adapting the format rather than the function of core components.
Training formats and materials, originally used in Canada, were adapted by local trainers, in consultation with the international trainers and local adaptation teams, consisting of local healthcare and wellbeing organizations. For example training content on medical assistance in dying needed to be adapted to align with local end-of-life care legislation. These adaptations were part of a broader adaptation process to tailor the Canadian Nav-CARE© program for implementation across six different European countries and is in depth described and evaluated by Van Campe et al. [22].
**Development**,** goal and learning methods of the Train-the-Trainer program**
**Development**: In February 2022, two international trainers, including experienced trainers from the Nav-CARE© program, collaborated to develop the Train-the-Trainer program for the EU NAVIGATE project and determine the learning strategies. **Goal**: The Train-the-Trainer program aimed to familiarize the local trainers with the program content, key navigator competencies, and how to facilitate the training for the navigators within their setting and local context and how to cooperate with and support the coordinator. It included a Train-the-Trainer week (held in Brussels, April 2022, with international and local trainers from the six countries). The Train-the-Trainer week schedule can be found in Appendix 1. Additionally, there were online monthly follow-up sessions with the local trainers led by one international trainer to facilitate ongoing support and knowledge sharing.
**Learning methods**: Both didactic and interactive methods and materials were used, with a particular emphasis on interactive learning. The approach aimed to encourage mutual learning, open communication, and constructive feedback. For example, local trainers were asked to prepare a training module independently in advance, which was then discussed and refined with feedback from other local trainers and the international trainers during the Train-the-Trainer week.[19–21]

## Methods

### Study design

We evaluated the Train-the-Trainer program to implement a navigation program for older people with cancer and their families, using multiple perspectives and data collection methods informed and retrospectively structured by the Kirkpatrick model [9]. This model proposes four levels of evaluation: Reaction, Learning, Behavior, and Results. Table 1 shows how we applied the Kirkpatrick model, the research questions, timing, and data collection methods used for the evaluation.

Although the evaluation was not initially designed to use the Kirkpatrick model, we adopted the model during the analysis phase to structure and interpret the data collected for the process evaluation of the navigation program. After mapping existing data onto its four levels, we conducted additional interviews with trainers to address potential gaps.

### Participants

There were four types of participants in this study: international trainers, local trainers, navigators, and older people with cancer and declining health (Fig. 1). The international trainers included one experienced trainer working for the EU NAVIGATE project (also the local trainer in Belgium), and one developer and experienced trainer from the Nav-CARE© program. Local trainers were recruited in the participating countries based on a profile description and key criteria determined by the international trainers (Appendix 2). During the implementation period of the navigation program, some local trainers were replaced due to job transitions or maternity leave. In these cases, the original trainer provided training to their successors, and each new trainer participated in a brief online meeting with the international trainer to ensure continuity and clarity.

Navigators were recruited in different ways according to the best fit within the context of the local setting in each country, e.g., via existing volunteer organizations in health or palliative care, websites, advertisements on local radio or paper (see recruitment paper [23] for more details). Navigators were not required to have certain qualifications or background to be eligible, but were thoroughly screened by professionals before intake. Older people (70 years or above) with an active cancer diagnosis regardless of stage or treatment and in declining health, scored on the Clinical Frailty Scale (at least 1 change in CFS ending in score 4 in the last 6 month or scoring 5 or higher), were recruited through hospitals, via self-referral, general practitioners, and community-based health organizations (see evaluation protocol [13] for details). All participants that received navigation support for at least six months, were included for analysis in this study.

Approvals from the relevant ethics committees for this study were obtained in all participating countries, in accordance with the Declaration of Helsinki (see ethics declaration). All participants gave written informed consent prior to data collection. All obtained data were pseudonymized.

### Data collection

We operationalized each level of the Kirkpatrick model (Reaction, Learning, Behaviour, Results) as shown in Table 1, and collected data accordingly. Participant characteristics were assessed through a short questionnaire included in the other surveys, outlined below.

To assess Reaction and Learning (levels 1 and 2), we conducted a short survey with the local trainers immediately after the Train-the-Trainer week. The survey contained fifteen questions, on a five-point Likert scale (Totally disagree – 1, Disagree a bit − 2, Neither agree nor disagree − 3, Agree a bit − 4, Totally agree − 5). Questions assessing level 1 focused on local trainers’ perceived relevance of the training, as well as their satisfaction and engagement with it. Questions assessing level 2 focused on local trainers’ knowledge, skills, attitude, confidence, and commitment.

To assess Behaviour (level 3), local trainers filled in an online structured diary on a weekly basis for approximately two years. Questions included, but were not limited to, “Which activities did you spend time on this week?” or “Which successes or challenges did you encounter during these activities?” or “On which of the activities you mentioned did you spend most time this week and how many hours approximately?”.

To assess Results (level 4), navigators completed a short questionnaire immediately after the training focusing on their understanding of key competencies and their confidence in applying them. This survey contained ten questions on a five-point Likert scale (Totally disagree – 1, Disagree a bit − 2, Neither agree nor disagree − 3, Agree a bit − 4, Totally agree − 5). Questions included, for example, “I understand what is expected from me to address the quality-of-life concerns of older people with cancer and their families” and “I feel confident in my ability to address the quality-of-life concerns of older people with cancer and their families”.

The older people with cancer and declining health included in this study completed a short questionnaire, part of a larger survey, after completing a trajectory of approximately 6 months with their navigator. The questionnaire consisted of five questions scored on a 5-point Likert scale (Totally disagree – 1, Disagree a bit − 2, Neither agree nor disagree − 3, Agree a bit − 4, Totally agree − 5), each asking about one of the five navigator competencies. Questions included, for example, “My navigator talks with me about what is important for me with regard to my quality of life”.

In addition, researchers (IBV & FVC) conducted two group interviews with the local trainers after two years of training and executing their role. Each local trainer participated in exactly one of the two interviews. The purpose was to [1] provide the opportunity for the local trainers to suggest refinements to the preliminary analysis or raise new insights not yet captured and [2] gain a deeper understanding of experiences with the Train-the-Trainer program, the ongoing support and their feeling of preparedness for their role and tasks after completing most of the trajectory. Researchers (FVC & IBV) developed the topic guide after analyzing the surveys and diaries (see Appendix 3). Example questions were: ‘This is the top five of most mentioned activities in the trainer diaries. If you look back at the past two years of you being trainer, does this top five accurately reflect your activities during this time?’; ‘Which aspects of the train-the-trainer week were most and least useful to you? In these group interviews, the local trainers could share, compare, and challenge each other’s perspectives, as well as discuss the preliminary results of prior data collection [24], providing a more comprehensive view over the trainer experience throughout the project.

After the group interviews, one researcher (FVC) conducted a semi-structured interview with one international trainer to reflect on the preliminary results of all prior data. The aim was to gain insights from their perspective and explore potential mechanisms that might explain the preliminary results.

### Data analysis

All quantitative data were examined through descriptive statistics, i.e. median with interquartile range, using SPSS Statistics 29.0.0. Qualitative data were analyzed using conventional qualitative content analysis, a method in which coding categories are derived directly from the raw data, without preconceived theoretical frameworks [25]. This approach is particularly suitable for exploring phenomena where existing theory or research literature is limited. Microsoft Excel was used as an organizational tool to support the coding process for the data of the diaries. Two researchers (FVC & IBV) independently assigned open codes to the diary entries, then compared and grouped these codes into subcategories and main categories until consensus was reached. The question, “Which of the activities did you spend the most time on this week, and approximately how many hours?”, was analyzed descriptively. The reported hours were summed, and percentages were calculated by dividing the hours spent on each activity by the total number of reported hours. The resulting categories informed the topic guide for the subsequent group interviews, enabling a member check, during which local trainers could confirm, elaborate on, or contest the categories [26]. The parts of interview transcripts concerning this member check were analyzed using narrative analysis, considered as the most appropriate approach due to its focus on the experiencers and meaning-making process of the trainers [27]. The transcripts of the second part of the group interviews with the local trainers and the semi-structured interview with the international trainer were examined using conventional qualitative content analysis [25] with MAXQDA by the researcher (FVC). The researcher (FVC) performed open coding, categorized into (sub)categories and discussed abstraction into main categories within a team (KC, LVdB, TS), until consensus was reached [25].

**Table 1:**
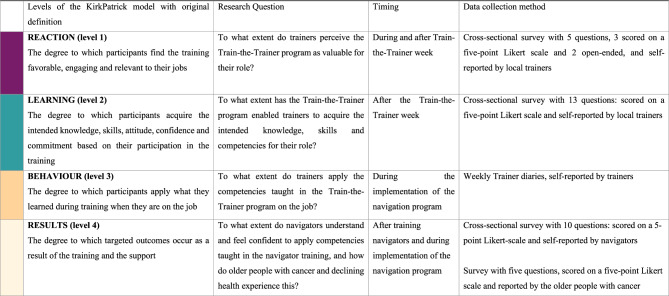
overview of the evaluation framework: KirkPatrick levels, research questions, timing, and data collection methods

## Results

### Participants

A total of 1 international trainer, 11 local trainers, 155 navigators and 101 older people with cancer and declining health were involved in the evaluation of the Train-the-Trainer program (Table 2). Most participants across trainers (83,3%), navigators (70%) and older people with cancer (58%) were female. Most trainers were aged between 30 and 49 years, most navigators between 50 and 69 years and all older people with cancer and declining health were aged 70 or above. Most navigators were from Portugal (27%) and Italy (23%), and most older people with cancer were from Portugal (20%) and Belgium (19%). Across the study period, 6 trainers started the Train-the-Trainer program and all completed, but during implementation some trainers dropped-out and were replaced (Table 2), 161 navigators started the training and 155 completed the training. All older people with cancer and declining health (*n* = 101) received the navigation program for at least 6 months.

**Table 2:** Characteristics of participants in the evaluation of the Train-the-Trainer program

Characteristics	Trainers*				Navigators			Older people with cancer and declining health
Sample size per level of the Kirkpatrick model	*n* = 6 Cross-sectional surveys Level 1 & 2	*n* = 11 Local Trainer diaries Level 3	*n* = 6 Group interview trainers (across all levels)	*n* = 1 Interview international trainer (across all levels)	*n* = 155 (%) Cross-sectional survey Level 4			*n* = 101 Survey Level 4
Sex								
Female	5	9	5	1		109 (70%)	58 (58%)	
Male	1	2	1	-		46 (30%)	42 (42%)	
Age								
18–29 years	1	2	2	-		13 (8%)	-	
30–49 years	3	8	4	1		34 (21%)	-	
50–69 years	2	1	-	-		86 (55%)	-	
70 + years	-	-	-	-		22 (14%)	101 (100%)	
Participating Country								
Belgium	1	2	1	-		28 (18%)	19 (19%)	
Ireland	1	1	1	-		27 (17%)	18 (18%)	
Italy	1	3	1	-		36 (23%)	14 (14%)	
Netherlands	1	1	1	-		14 (9%)	12 (12%)	
Poland	1	1	1	-		9 (6%)	18 (18%)	
Portugal	1	3	1	-		41 (27%)	(20%)	

-=Not applicable, *trainer-related data are divided into four columns because the sample size for data collection instruments and time points differs, due to factors such as job changes, maternity leave, and other circumstances during implementation (± 2 years) of the Train-the-Trainer program

## Level 1 reaction and level 2 learning

**Fig. 2:**
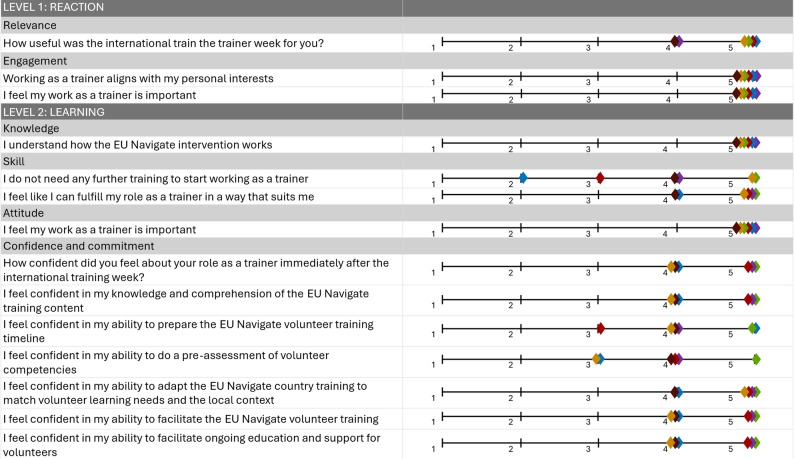
Level 1 Reaction and Level 2 Learning: relevance, engagement, knowledge, skill, attitude, confidence and commitment scores* reported by local trainers (*n* = 6). *the colored rectangles indicate the responses of the individual trainers scored on a 5-point Likert scale (Totally disagree – 1, Disagree a bit − 2, Neither agree nor disagree − 3, Agree a bit − 4, Totally agree − 5)

Based on the descriptive analysis of the questionnaires completed by local trainers from the six participating countries (Fig. 2), the Train-the-Trainer week was rated highly useful, with a median score of 5, “totally agree” (IQR = 0.75). All trainers “totally agreed” that working as a trainer aligned with their personal interests and that their work was important. Regarding learning outcomes, all trainers “totally agreed” on understanding how the navigation program works. The item “I do not need any further training to start working as a trainer” had a median score of 4, “agree” (IQR = 1.5). Trainers rated their ability to fulfill the trainer role in a way that suits them, with a median score of 5, “totally agree” (IQR = 0.75). Confidence-related items had median scores ranging from 4 (agree) to 5 (totally agree), with IQRs between 0.75 and 1.

.

In the group interviews with the local trainers, we identified the following categories that facilitated their learning process. Getting the time to *grasp the project and the trainer role* during the Train-the-Trainer week facilitated their learning process to get a clearer picture of navigation as a concept, the structure and aims of the program, and their responsibilities within the navigator training, as illustrated in the following quote by one of the local trainers: “It was probably the first time that we really got deeply into EU NAVIGATE and what to do practically with the volunteers because before we had like a generic concept.” Local trainers highlighted that *getting to know the team* and fostering *enthusiasm for the project and their trainer role* also facilitated future cooperation, and that meeting both international trainers and other local trainers from diverse backgrounds was helpful in “creating a bond”. All local trainers reported feeling confident and ready to begin their tasks: “I became very enthusiastic also about the program and also about being a trainer, […] when I was leaving, I was really much excited that I would be a trainer within this project and we felt confident in starting our role.” In the one-to-one interview with one of the international trainers, she explained that “the trainers got a lot of time to make the navigator training their own and this feeling of ownership often makes one feel confident.”

## Level 3 behavior

### Reported activities by the local trainers and member check

**Fig. 3:**
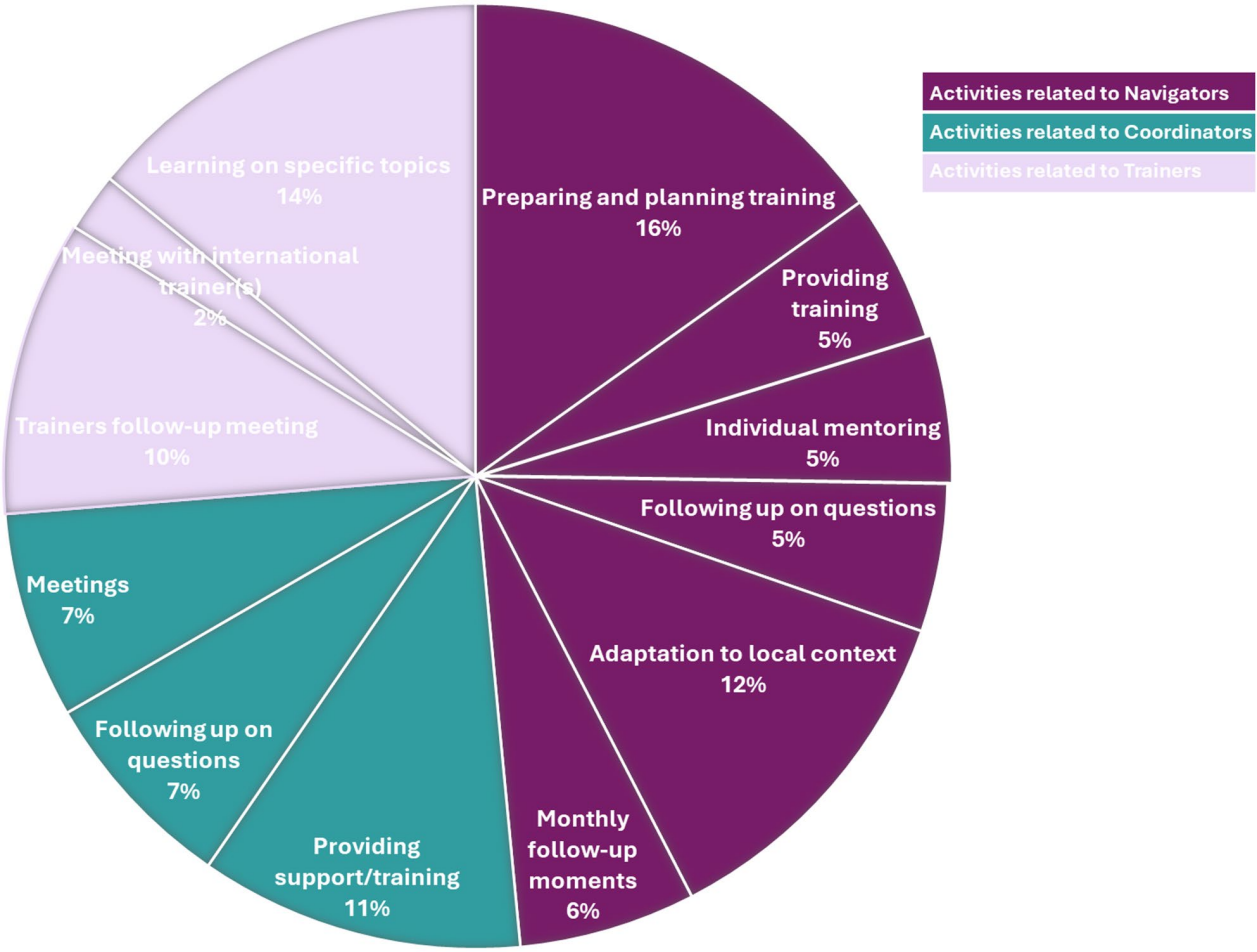
Overview of time-distribution of direct competency-related activities reported by local trainers in weekly diaries for approximately two years

The analysis of the weekly trainer diaries indicated that local trainers applied the competencies taught in the Train-the-Trainer program during the implementation of the navigation program (Fig. 3). Most time was spent on preparing and planning training (16%), and least time was spent on meeting with international trainer(s) (2%).

During the group interviews, all local trainers agreed with the overview of direct competency-related activities and confirmed the time distribution. A key point raised in both group interviews was that the time spent on certain activities shifted over the course of the program. At first, more time was dedicated to planning and preparing training sessions, while later phases required more focus on navigator support and mentoring.

### Reported successes and challenges in applying competencies by the local trainers

The weekly trainer diaries yielded a total of 145 entries describing competency-related successes. These included: receiving positive feedback from navigators (30 entries), such as their “enthusiasm about the project”, “their trajectory with the client”, or about the support they received. Other successes mentioned were: successful adaptation (25 entries), delivery of the training (23 entries), and the facilitation of follow-up support for the navigators (29 entries), particularly when follow-up meetings were perceived as useful and when “navigators were observed applying the competencies they had learned”.

During the member check, trainers agreed with the identified categories of success but not with their prioritization. The most important success, according to all trainers, was the ability to adapt the training to local contexts. Most trainers described this process of adaptation as both their greatest success and their most significant challenge. Identifying where adaptation is needed was challenging. However, the ability to adapt directly in practice improved the quality of the training, as it allowed the content to be better aligned with the actual training needs, while still adhering to the targeted competencies. For example, in Italy, the navigation model was a new concept, especially “the idea of the navigator as an advocate for patient empowerment”, something not commonly practiced in the Italian healthcare system. The trainer found it a success to be able to put more emphasis on this advocacy role competency. In Poland, where navigators were social workers accustomed to primarily “fixing practical issues”, the ability to adapt the training enabled the trainer to place greater emphasis on emotional support.

The trainers’ weekly diaries yielded a total of 85 entries of competency-related challenges. These included time constraints and workload (22 entries), often due to “balancing multiple roles within the project” or all tasks that converge within the same week, which creates a sudden accumulation of workload. Another commonly mentioned challenge was limited collaboration within country teams (17 entries), attributed to factors such as “poor communication”, “unclear responsibilities”, or understaffing. Trainers also faced difficulties in adapting training materials (13 entries) and planning sessions (10 entries), particularly when dealing with low attendance of navigators or “practical barriers”.

During the member check, trainers agreed with the identified challenges and their ranking, but added two more. The first concerned the role of emotional support. Navigators with longer navigation trajectories indicated that simply being present and listening was often central to the experience of older people with cancer and declining health. Trainers recognized this challenge and integrated a stronger emphasis on emotional support and relational aspects in later training sessions. Second, the module on supporting technology use was seen as less relevant in the European context. Trainers reported low digital literacy among navigators and limited digital needs among older participants, leading many to adapt, postpone, or exclude this module from their training programs.

### Barriers and facilitators to the application of competencies

In the group interviews we identified the following categories that facilitated or formed a barrier to apply the competencies. *Peer support* was frequently mentioned as a facilitator, with trainers appreciating the possibility to “count on people if you had a question or just needed a chat.” The follow-up meetings were seen as useful opportunities to *exchange experiences and knowledge* and to learn from one another. The *availability and expertise of the international trainers* also facilitated their job. As one local trainer explained “They were a really good source of information because they are so experienced themselves and warm personalities, so that was really helpful.” Some trainers noted that the *online format of the follow-up meetings* worked as a barrier, since they felt less engaged. There was shared agreement that in-person meetings during implementation would be more valuable, helping to maintain engagement and support ongoing learning. As the international trainer explained, “this would be of added value if the navigation program continues in each setting, to recalibrate and also see pieces of each other’s training and exchange good practices.”

## Level 4 results

### Navigator understanding and confidence in applying competencies

**Fig. 4:**
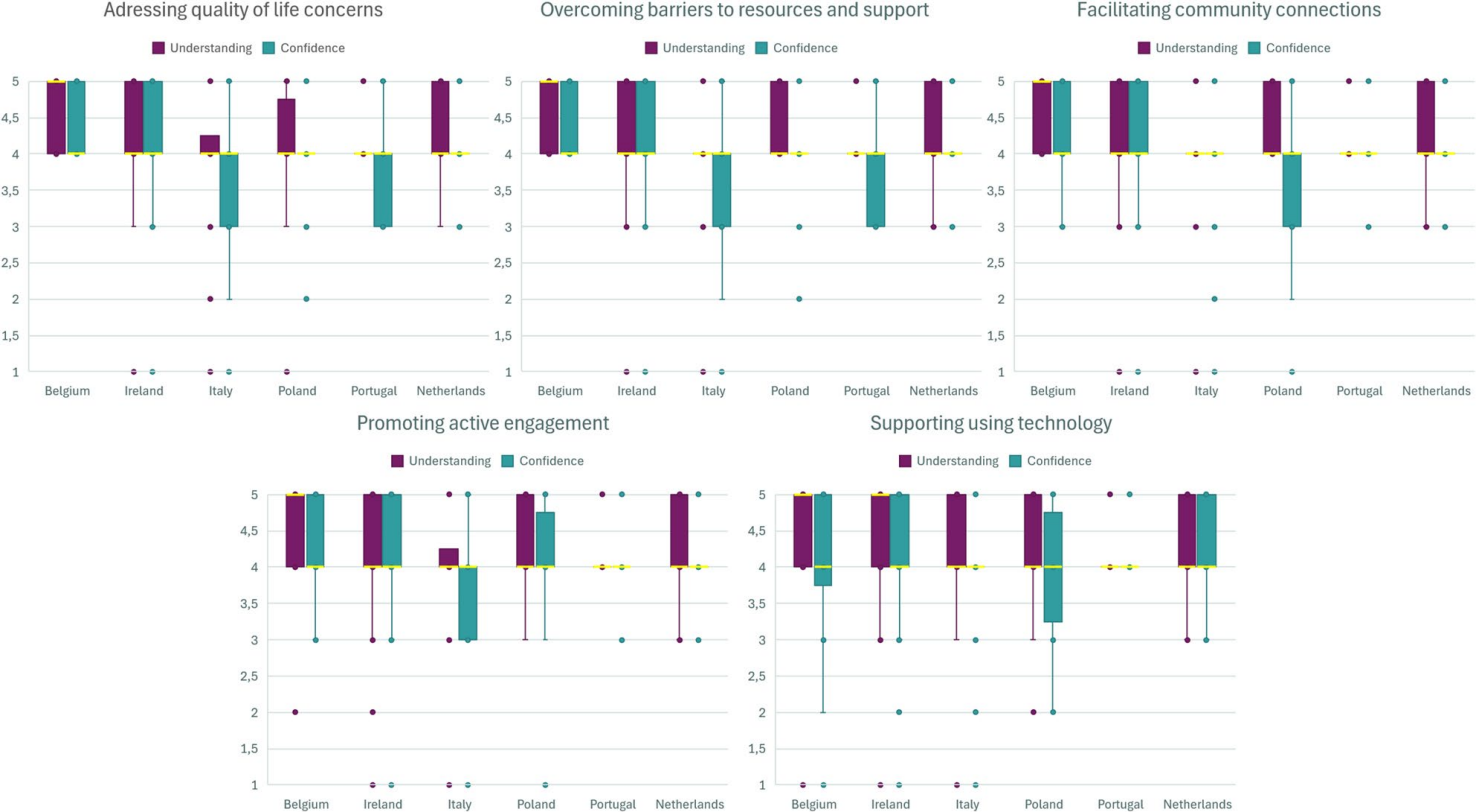
Navigator understanding and confidence median and interquartile range per competence per country. *scored on a 5-point Likert scale (Totally disagree – 1, Disagree a bit − 2, Neither agree nor disagree − 3, Agree a bit − 4, Totally agree − 5). Median emphasized by horizontal yellow line

Across all participating countries, the median overall understanding and confidence scores reported by all navigators (*n* = 155) was 4 (IQR = 1) and 4 (IQR = 1) respectively on a 5-point Likert scale (1 = Totally disagree, 5 = Totally agree). Figure 4 displays boxplots that outline median and interquartile range per competence per country.

### Experiences of older people with cancer and declining health

**Fig. 5:**
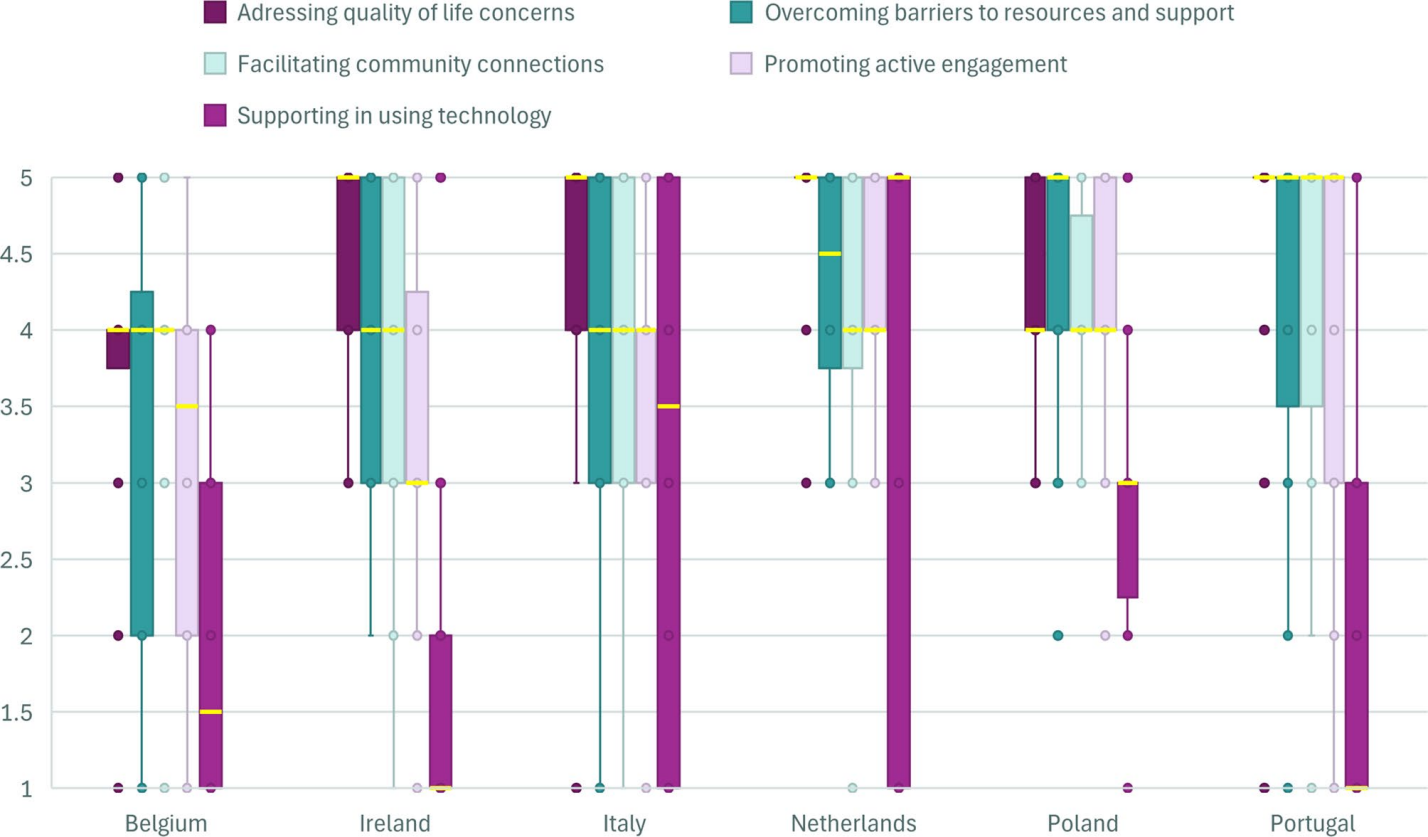
Median* and interquartile range reported by older people with cancer and declining health of application of navigator competencies per country. *scored on a 5-point Likert scale (Totally disagree – 1, Disagree a bit − 2, Neither agree nor disagree − 3, Agree a bit − 4, Totally agree − 5). Median emphasized with horizontal yellow line. Participants were asked to which extent they (dis)agree on whether their navigator talks or supports them per statement, which are linked to the taught competencies. For example, my navigator talks to me about what is important for my quality of life/ limits my quality of life

Participants (*n* = 101) across all six countries reported that navigators generally addressed the intended competencies of the program, with median scores between 1 (IQR = 2) and 5 (IQR = 1) across competencies scored on a 5-point Likert scale (1 = Totally disagree, 5 = Totally agree). Figure 5 displays boxplots that outline median and interquartile range per competence per country.

## Discussion

### Main findings

The results from this multi-method multi-level and multi-perspective evaluation involving 257 participants (local and international trainers, navigators and beneficiaries of the navigation program) indicate that the Train-the-Trainer program, in context of the EU NAVIGATE project, was positively evaluated in terms of its learning outcomes and trainer experiences. The Train-the-Trainer program has consistently favorable outcomes across the different levels and implementation settings, despite the specific contextual challenges. Local trainers reported that the Train-the-Trainer week was highly useful, reporting strong alignment with their role, clear understanding of the program, and high confidence scores. They felt prepared for their role and to implement the training in their local setting. Local trainers applied the competencies taught in the Train-the-Trainer program during the implementation of the navigation program. Most of their time was spent on training preparation, topic-specific learning, local adaptation, and navigator support. Peer support and exchanging experiences and knowledge facilitated applying the competencies taught during the training. Local trainers highlighted that they appreciated the continuous support and expertise of the international trainers. Navigators reported a high understanding and confidence in applying competencies, with highest scores for addressing quality of life and overcoming barriers, and lower scores for supporting technology use. Older adults with cancer responded that navigators mostly addressed quality of life concerns, supported them in overcoming barriers to resources and support, and facilitated community connections.

Even though the Train the Trainer program, navigator training, and the navigation program were implemented in diverse health- and social-care settings, and with different types of navigators (e.g., volunteers and social workers), patterns across countries and evaluation levels appeared broadly similar, with no clear indications of systematic differences based on the descriptive data. These minor differences in results across countries may be explained by the fact that we started from a strong common framework, based on prior work within Nav-CARE© and the flexible approach taken during this Train-the-Trainer program. The first reason may be that the international trainers focused on taking time to get to know each other and encouraged peer support and constructive feedback among the local trainers. This created a safe space to work in, with opportunities for exchanging experiences and knowledge. Other studies evaluating Train-the-Trainer programs in public health or end-of-life care indicate as well that peer-to-peer approaches in combination with expert facilitation are essential principles in order to successfully disseminate knowledge and skills via a Train-the-Trainer model [28, 29].

Secondly, the international trainers allowed adaptation of the format and content of the navigator training, as long as the training addressed the same core functions of the competencies. We used the ADAPT guidance [21] to guide this adaptation process. Identifying where adaptation is needed was challenging. For example, deciding whether practice example cases or role-plays could remain in their original form or needed contextual tailoring to resemble realistic local scenarios required substantial discussion among international trainers. However, adaptation was necessary to align with local training needs, due to differences in setting or context. The ability to adapt improved the quality of the training according to the local trainers. Just as in this study, navigation programs exist in the continuum of care, are taking place in various settings with potentially different contexts (i.e. socio-economic, legal, epidemiological, political, geographical and epidemiological), and with different types of navigators (i.e. volunteers and social workers). Therefore, systematic adaptation of navigator training to local health system needs and older adults’ care priorities is recommended. Similar research on the learning outcomes and training experiences within a Train-the-Trainer program in primary care and public health aligns with these findings, stating that the success of the Train-the-Trainer model depends on contextual adaptation or allowing the courses to be tailored to local issues [30]. Being continuously supported by peers and experts in combination with the ability to contextually adapt the training contributed to a sense of ownership and feeling prepared for the trainer role in a way that suited each trainer.

### Strengths and limitations

To our knowledge, this is the only study that provides an in-depth evaluation of Train-the-Trainer program for a navigation program. Our international, multi-method evaluation, conducted over an extended period, and using the four levels of the Kirkpatrick model supported a comprehensive analysis of the Train-the-Trainer program. It captured perspectives from all key stakeholders involved: international and local trainers, navigators, and older people with cancer and declining health (program beneficiaries), which aims to support credibility.

One limitation is that the study was not originally designed based on the Kirkpatrick model, which might have led to gaps in the evaluation process. For example, for level 4 the beneficiaries of the program (older people with cancer) were asked whether tasks that relate to the competences were performed. If the older person with cancer did not have the need for that task, such as support in using technology, than the older person with cancer will most likely not have indicated that this tasks was performed, even though the navigator might have the competence. It might be that this competence was less needed, instead of well-trained. Thirdly, quantitative data were self-reported, reflecting participants’ perceptions of their learning and the program’s impact. It is possible that respondents either overestimated or underestimated their understanding or confidence when completing the questionnaires. Lastly, we would like to recognize that there might be social desirability bias among respondents.

### Conclusion and implications for research, policy and practice

Growing evidence suggests that Train-the-Trainer programs can effectively expand the reach and capacity to successfully disseminate knowledge and skills [16, 17]. This study adds to the literature on the potential role of Train-the-Trainer programs as an implementation strategy to strengthen training capacity and program reach to support age-friendly health system development, such as the integration of navigation programs for older people with cancer in Europe.

Further research is needed to examine the specific contribution of Train-the-Trainer approaches to service delivery outcomes and to the sustainability of age-appropriate health programs, such as navigation programs. While Train-the-Trainer programs have demonstrated cost-effectiveness and potential to enhance sustainability in various health domains [18, 28], their impact within the context of navigation programs, particularly in European settings, remains underexplored. This evaluation contributes to addressing that gap but future studies should examine how best to adapt training content to local contexts and how this contributes to the long-term sustainability. In this study, we were informed by the ADAPT guidance [21], which emphasizes adapting the format rather than the function of core components. This may provide a useful framework for future research on contextual adaptation in training design. The lack of consensus on core competencies for navigators, both in Europe and internationally, further underscores the need for continued research [8].

Navigation programs are gaining attention in Europe and have shown promising results [3, 5]. Policy-makers should consider investing in Train-the-Trainer programs for navigation programs as part of broader strategies to strengthen long-term age-appropriate support systems. Policy support is needed to develop clear standardized guidelines and frameworks for training navigators, for example on how to systematically adapt navigation trainings or define core competencies [5].

Although volunteer training in oncology has existed in Europe for some time, the concept of navigation and the various navigation models are still relatively new. This evaluation and its insights may inspire others seeking to evaluate similar training programs for complex interventions and inform strategies for implementing training for navigation programs more broadly. Our findings highlight that contextual adaptation is essential for successful delivery. Practitioners implementing similar interventions should be aware that adapting training to specific local needs, while maintaining core functions, is a necessary condition for effective and sustainable implementation. While we recognize that the Train-the-Trainer program is not the only factor for successful outcomes of navigation programs, it appears to be an important precondition.

This method and the insights can inspire others seeking to evaluate similar Train-the-Trainer programs for complex interventions and might inform future strategies for implementing training for navigation programs more broadly.

## Electronic Supplementary Material

Below is the link to the electronic supplementary material.

Supplementary Material 1: Appendix 1: Train-the-Trainer week schedule.

Supplementary Material 2: Appendix 2: Profile description and key criteria for recruitment local trainers.

Supplementary Material 3: Appendix 3: Topic guide group interview with local trainers.

## Data Availability

The datasets used and/or analyzed during the current study are available from the corresponding author on reasonable request.

## Declarations

### EU NAVIGATE Collaborative Author List and full list of consortium research members

Rose Miranda^1^, Chelsea Vinckier^1^, Shannen De Vos^1^, Chiara Verschaeren^1^, Eline Naert^2^, Helena Du Cheyne^2^, Roeline Pasman^3^, Annicka van de Plas^3^, Gloria Puurveen^4^, Ashford Kelly^4^, Ilona Barańska^6^, Kasia Slaby^6^, Agata Stodolska^6^, Emilia Malmur^6^, Natalia Witek^6^, Maja Furlan de Brito^7,8^, Vitor Rodrigues^7^, Sonia Silva^7^, Inês Correia^7^, Sandra Gervasio^7^, Peter May^9^,Amanda Lavan^9^, Gemma O’Keeffe^9^, Siofra Hearne^9^, Monica Gandelli^10^, Chiara Lusetti^10^, Laura Gangeri^11^, Bianca Scacciati^11^.

^1^Department of Family Medicine and Chronic Care, Vrije Universiteit Brussel, Brussels, Belgium.

^2^Department of Public Health and Primary Care, Universiteit Gent, Ghent, Belgium.

^3^Amsterdam UMC, Vrije Universiteit Amsterdam, Department of Public and Occupational Health, Expertise Center for Palliative Care Amsterdam UMC, Amsterdam, Netherlands.

^4^University of British Columbia Okanagan, Kelowna, BC, Canada.

^5^University of Alberta, Edmonton, Alberta, Canada.

^6^Laboratory for Research on Aging Society, Epidemiology and Preventive Medicine, Medical Faculty, Jagiellonian University Medical College, Kraków, Poland.

^7^Faculty of Medicine, University of Coimbra, Coimbra, Portugal.

^8^Cicely Saunders Institute of Palliative Care, Policy & Rehabilitation, King’s College London, London, UK.

^9^Trinity College Dublin, Dublin, Ireland.

^10^Lega Italiana per la lotta contro i tumori di Milano, LILT Milano Monza Brianza, Milano, Italy.

^11^ Clinical Psychology Unit, Fondazione IRCCS Istituto Nazionale dei Tumori, INT, Milano, Italy.

## References

[1] Valaitis RK, Carter N, Lam A, Nicholl J, Feather J, Cleghorn L. Implementation and maintenance of patient navigation programs linking primary care with community-based health and social services: a scoping literature review. BMC Health Serv Res. 2017;17(1):1–14. 10.1186/s12913-017-2046-1 . PubMed PMID: 28166776. 28166776 10.1186/s12913-017-2046-1 PMC5294695

[2] Wells KJ, Valverde P, Ustjanauskas AE, Calhoun EA, Risendal BC. What are patient navigators doing, for whom, and where? A national survey evaluating the types of services provided by patient navigators. Patient Educ Couns. 2018;101(2):285–94. 10.1016/j.pec.2017.08.017. 28935442 10.1016/j.pec.2017.08.017 PMC5808907

[3] Chan RJ, Milch VE, Crawford-Williams F, Agbejule OA, Joseph R, Johal J, et al. Patient navigation across the cancer care continuum: An overview of systematic reviews and emerging literature. CA Cancer J Clin. 2023;73(6):565–89. 10.3322/caac.21788 . PubMed PMID: 37358040. 37358040 10.3322/caac.21788

[4] Budde H, Williams GA, Winkelmann J, Pfirter L, Maier CB. The role of patient navigators in ambulatory care: overview of systematic reviews. BMC Health Serv Res. 2021;21(1). 10.1186/s12913-021-07140-6 . PubMed PMID: 34706733. 10.1186/s12913-021-07140-6 PMC8555047 34706733

[5] Budde H, Williams GA, Scarpetti G, Kroezen M, Maier CB. What are patient navigators and how can they improve integration of care? HEALTH SYSTEMS AND POLICY ANALYSIS [Internet]. 2022. Available from: https://doi.org/www.euro.who.int. 35129934

[6] Kokorelias KM, Shiers-Hanley JE, Rios J, Knoepfli A, Hitzig SL. Factors Influencing the Implementation of Patient Navigation Programs for Adults with Complex Needs: A Scoping Review of the Literature. Health Services Insights. SAGE Publications Ltd; 2021. 10.1177/11786329211033267. 10.1177/11786329211033267 PMC8287353 34349519

[7] Liu S, Tang W, Chang Q, Lei J, Yue H, Hou L, et al. Implementation and Evaluation of a Training Program to Improve Patient Navigators’ Competencies: A Quasi-Experiment at a Public Tertiary Hospital in China. Healthc (Switzerland). 2025;13(4). 10.3390/healthcare13040387. 10.3390/healthcare13040387 PMC11855021 39997263

[8] Ustjanauskas AE, Bredice M, Nuhaily S, Kath L, Wells KJ. Training in Patient Navigation: A Review of the Research Literature. Health Promot Pract. 2016;17(3):373–81. doi:10.1177/1524839915616362 PubMed PMID: 26656600. 26656600 10.1177/1524839915616362 PMC4899310

[9] Kirkpatrick JD, Kirkpatrick WK. Kirkpatrick’s Four Levels of Training Evaluation. Association fot Talent Development; 2016.

[10] Desilets LD. An update on Kirkpatrick’s model of evaluation: Part two. J Contin Educ Nurs. 2018;49(7):292–3. 10.3928/00220124-20180613-02 . PubMed PMID: 29939374. 29939374 10.3928/00220124-20180613-02

[11] Gökhan Ulum Ö. Program Evaluation through Kirkpatrick’s Framework [Internet]. Hakkari; 2015 Jul. Available from: https://www.researchgate.net/publication/295899652

[12] Kavulya JM. Evaluation of Training Programmes: A Review of Selected Models and Approaches [Internet]. 2021. Available from: https://www.finessejournals.com

[13] Smets T, Pivodic L, Miranda R, Van Campe F, Vinckier C, Pesut B, et al. Implementation and evaluation of a navigation program for people with cancer in old age and their family caregivers: study protocol for the EU NAVIGATE International Pragmatic Randomized Controlled Trial. Trials. 2024;25(1):800. 10.1186/s13063-024-08633-5. 39605055 10.1186/s13063-024-08633-5 PMC11603903

[14] Miranda R, Smets T, Pivodic L, Chambaere K, Pesut B, Duggleby W, et al. Adapting, implementing and evaluating a navigation intervention for older people with cancer and their family caregivers in six countries in Europe: the Horizon Europe-funded EU NAVIGATE project. Palliat Care Soc Pract. 2024;18. 10.1177/26323524241288873. 10.1177/26323524241288873 PMC11492236 39435050

[15] Pesut B, Duggleby W, Warner G, Ghosh S, Bruce P, Dunlop R, et al. Scaling out a palliative compassionate community innovation: Nav-CARE. Palliat Care Soc Pract. 2022;16. 10.1177/26323524221095102. 10.1177/26323524221095102 PMC9112317 35592240

[16] Pearce J, Mann MK, Jones C, van Buschbach S, Olff M, Bisson JI. The Most Effective Way of Delivering a Train-the-Trainers Program: A Systematic Review. J Continuing Educ Health Professions. 2012;32(3):215–26. 10.1002/chp.21148. 10.1002/chp.21148 23173243

[17] Poitras ME, Bélanger E, Vaillancourt VT, Kienlin S, Körner M, Godbout I, et al. Interventions to Improve Trainers’ Learning and Behaviors for Educating Health Care Professionals Using Train-the-Trainer Method: A Systematic Review and Meta-analysis. J Continuing Educ Health Professions. 2021;41(3):202–9. 10.1097/CEH.0000000000000375. 10.1097/CEH.0000000000000375 34292260

[18] Triplett NS, Sedlar G, Berliner L, Jungbluth N, Boyd M, Dorsey S. Evaluating a Train-the-Trainer Approach for Increasing EBP Training Capacity in Community Mental Health. J Behav Health Serv Res. 2020;47(2):189–200. 10.1007/s11414-019-09676-2 . PubMed PMID: 31898144. 31898144 10.1007/s11414-019-09676-2

[19] Duggleby W, Pesut B, Cottrell L, Friesen L, Sullivan K, Warner G. Development, Implementation, and Evaluation of a Curriculum to Prepare Volunteer Navigators to Support Older Persons Living With Serious Illness. Am J Hospice Palliat Med. 2018;35(5):780–7. doi:10.1177/1049909117740122 PubMed PMID: 29129107. 10.1177/1049909117740122 29129107

[20] Duggleby W, Robinson CA, Kaasalainen S, Pesut B, Nekolaichuk C, MacLeod R, et al. Developing Navigation Competencies to Care for Older Rural Adults with Advanced Illness. Can J Aging. 2016;35(2):206–14. doi:10.1017/S0714980816000131 PubMed PMID: 27093177. 27093177 10.1017/S0714980816000131

[21] Moore G, Campbell M, Copeland L, Craig P, Movsisyan A, Hoddinott P, et al. Adapting interventions to new contexts-the ADAPT guidance. BMJ. 2021;374. 10.1136/bmj.n1679 . PubMed PMID: 34344699. 10.1136/bmj.n1679 PMC8329746 34344699

[22] Van Campe F, Chambaere K, Pivodic L, Gilissen J, Pesut B, Duggleby W, et al. Systematic adaptation of public health palliative care interventions across settings using ADAPT guidance: Methodological learnings from the EU NAVIGATE project. Palliat Med. 2025;39(4):460–72. 10.1177/02692163251320507. 40105050 10.1177/02692163251320507 PMC12084666

[23] Du Cheyne H, Miranda R, Smets T, Onwuteaka-Philipsen B, Pasman R, Pesut B, et al. Identifying facilitators, barriers, and strategies to optimize recruitment of volunteer navigators for implementing a navigation intervention for older people with cancer and their families: a mixed-method study embedded in the international EU Navigate pragmatic randomized controlled trial. Palliat Care Soc Pract. 2024;18:1–164. 10.1177/26323524241280174.

[24] Stewart D, Shamdasani P, Rook D. Focus Groups. 2455 Teller Road, Thousand Oaks California 91320 United States of America. SAGE Publications, Ltd.; 2007. 10.4135/9781412991841.

[25] Stevens Peter. Qualitative data analysis : key approaches. SAGE; 2023. p. 386.

[26] Lloyd N, Hyett N, Kenny A. To Member Check or not to Member Check? An Evaluation of Member Checking in an Interpretive Descriptive Study. Int J Qual Methods. 2024;23. 10.1177/16094069241301383.

[27] Smith B, Monforte J. Stories, new materialism and pluralism: Understanding, practising and pushing the boundaries of narrative analysis. Methods Psychol. 2020;2:100016. 10.1016/j.metip.2020.100016.

[28] Mayrhofer A, Goodman C, Smeeton N, Handley M, Amador S, Davies S. The feasibility of a train-the-trainer approach to end of life care training in care homes: An evaluation. BMC Palliat Care. 2016;15(1). 10.1186/s12904-016-0081-z . PubMed PMID: 26801232. 10.1186/s12904-016-0081-z PMC4724146 26801232

[29] Yarber L, Brownson CA, Jacob RR, Baker EA, Jones E, Baumann C, et al. Evaluating a train-the-trainer approach for improving capacity for evidence-based decision making in public health. BMC Health Serv Res. 2015;15(1). 10.1186/s12913-015-1224-2 . PubMed PMID: 26652172. 10.1186/s12913-015-1224-2 PMC4676893 26652172

[30] Poitras ME, Couturier Y, Morin A, Student P, Poirier MD, Vaillancourt VT et al. Optimizing registered nurses in primary care 41 Key Elements for Implementing a Train-the-Trainer Intervention for Registered Nurses and Social Workers in Primary Care. 2025 Mar. 10.12927/cjnl.2025.27553ResearchPapers. 10.12927/cjnl.2025.27553 40219744

