# Supplementary material for: Evaluating a Train-the-Trainer program to implement a navigation program for older people with cancer across six European countries, as part of the EU NAVIGATE project: a Kirkpatrick multi-method evaluation
Source: BMC Health Serv Res. 2026 May 21;26:987. doi: 10.1186/s12913-026-14717-6 (PMC13371289; doi:10.1186/s12913-026-14717-6)
Supplement: Supplementary file 1 — Supplementary Material 1: Appendix 1: Train-the-Trainer week schedule. [file 12913_2026_14717_MOESM1_ESM.docx]

**TOPIC guide**

| **Introduction** | **5 min** |
| --- | --- |
| - Thank you for attending - Introducing interviewer and observer (Iris and Fien) - Purpose of the research and explaining the group interview - Pointing out confidentiality and informed consent - Pointing out that we are interested in their trainer role, not necessarily other tasks they performed during the project. - We have a lot to discuss so it is really nice when you give examples but try to keep them as short as possible - We will be talking about successes and challenges, activities, evaluation of the training and preparedness, further implementation of the program   Example: You all are or have been a trainer for the NavCare-EU project, some for 2 years, some shorter. Some have stopped, some will stop and some will continue. In the upcoming hour I am interested in your experiences with being a trainer for this project over the past 2 years. | |
| **Topic: successes and challenges** | **20 min** |
| MIRO: Prepare overview successes and overview challenges, & function to be able to rank them  Write biggest challenges and successes down in Miro and make them slightly different colour | |
| - Your biggest success (and why?)=> please explain - Do others recognise this? - These were the successes that were identified through the diaries.   - Could you rank them? From what you experienced to be the biggest successes to what you experience as least important successes? - Your biggest challenge (and why)? => please explain - Do others recognise this? - These were the challenges that were identified through the diaries.   - Could you rank them? From what you experienced to be the biggest challenges to what you experience as a less important challenge? | |
| **Topic: activities** | **10 min** |
| MIRO: Prepare overview activities | |
| This is the top 5 of most mentioned tasks/activities in the trainer diaries. If you look back at the past two years of you being trainer, does this feel right? | |
| **Topic: evaluation training and mentoring & preparedness for role and tasks** | **15 min** |
| - Which aspects of the train the trainer week were most useful to you? - Which aspects of the train the trainer week were least useful to you? - Which aspects of the follow-up international trainers meetings were most useful to you? - Which aspects of the follow-up international trainers meetings were least useful to you? - Did you feel prepared for your role ? - What have you missed to fulfill your role? - In what ways did the international trainer support you? - What did you miss in the support of the international trainer? | |
| **Topic: further implementation** | **10 min** |
| - Which adjustments would you make to the training, if any, to improve implementation after the research project is over? - Imagine you could do it all over, what would you do differently or do the same as a trainer? | |
| **Closing questions** | **5 min** |
| - What is something that you have learned over the past two years, that you will take with you?   (What was the most rewarding moment of your career as a trainer in this project?) | |
