# Supplementary material for: Evaluating a Train-the-Trainer program to implement a navigation program for older people with cancer across six European countries, as part of the EU NAVIGATE project: a Kirkpatrick multi-method evaluation
Source: BMC Health Serv Res. 2026 May 21;26:987. doi: 10.1186/s12913-026-14717-6 (PMC13371289; doi:10.1186/s12913-026-14717-6)
Supplement: Supplementary file 2 — Supplementary Material 2: Appendix 2: Profile description and key criteria for recruitment local trainers. [file 12913_2026_14717_MOESM2_ESM.docx]

Appendix 2. Profile description of local Trainers

| **Aim of this profile description:**  This document is to guide PIs in each country in selecting/recruiting a Country Trainer and to guide them throughout the EU NAVIGATE project. The document has 3 parts: (1) Key criteria for appointment of country trainers; (2) Key responsibilities and (3) Person specification.  **Who’s this profile description for:**  The PI of each country.  **Recruitment and time of employment:** 1. The Country Trainer is hired part-time for 3 years. *Please consult your country’s budget for specifics*. 2. Country Trainers are hired and trained before the end of April 2023. *Please consult the project timeline.*  ***Sources***: PACE EU FP7 project, Nav-CARE materials, and consultation with Wendy Duggleby & Barbara Pesut. |
| --- |

**Part I: Key Criteria for appointment of EU NAVIGATE ‘Country Trainers’**

1. **Fluent in country language and good ability to speak English:** The international blended training that will be provided to the Country Trainers is predominantly provided in English. If Country Trainers are meeting with other country trainers, the dominant language will be English. Country Trainers, therefore, need to be able to understand and speak English.
2. **Previous experience in working with volunteers:** The Country Trainers must have *some* prior working experience in working with or as a volunteer in social or healthcare settings.; is supported by someone who has trained or worked with volunteers before.
3. **Healthcare background, ideally in palliative, nursing, social, gerontology, mental health or oncology care:** The Country Trainer has a social or healthcare degree (i.e., Nursing, Social Work, Psychology, Sociology, …) and has working experience in a palliative, nursing, social, gerontology, mental health or oncology care setting (inpatient or outpatient).
4. **Knowledge of the local/regional healthcare system in which the Nav-CARE intervention is to be implemented/tested. Ideally also has knowledge about the existing health and social care services in that region:** It is important that the Country Trainer has a good knowledge/understanding of the healthcare system in the region, to properly teach the navigators about the healthcare system and healthcare options. Ideally, he/she has good knowledge of the existing health and social care services in the region. The patients, living with cancer at home, often have other needs beside the medical needs. Therefore, a navigator needs the have a good knowledge of the local social care/community services as well needs to have a good knowledge of the general social organizations/activities in communities.
5. **Able to talk openly about death and dying**: The Country Trainer is able to speak openly about death and dying, in order to facilitate a conversation about (supportive and) palliative care with the patient and caregiver.
6. **Ability to train, empower and motivate coordinators and volunteers:** The EU NAVIGATE intervention is about training and empowering volunteers, so that they feel comfortable in navigating older persons with cancer. The Country Trainer needs to be able to teach, train and motivate people.
7. **Ability to create a participatory learning environment for volunteers:** Since the Country Trainers will be teaching adult volunteers, it is important that he/she can adapt the trainings depending on the background, previous experience and diversity of the volunteers. Using the input, questions and experiences of the volunteers is important to create a good training.

**Part II: KEY RESPONSIBILITIES**

***Assist in the adaptation of the Nav-CARE intervention:***

- If already hired, it is highly recommended that the Country Trainer attends the Local Adaptation Team meetings, and is involved in the cultural adaptation and feasibility review of the Nav-CARE materials.
- Review all Nav-CARE materials
- Assist local partners (which will engage in implementing the Nav-CARE intervention) to construct a Local Resource Guide.
- Take the lead on adapting and developing the training that will be provided to local navigation coordinators and navigators.

***Continuous communication with Local Partners, Navigation Coordinators and Navigators:***

- Get to know and build a good relationship with the volunteer organizations/local partners and the volunteer/ navigation coordinators.
- Identify organizational needs of local partners.
- Empower and motivate volunteer/navigation coordinators and support them in their tasks.
- Be available throughout the implementation of Nav-CARE to support and coach navigation coordinators and support them in supporting their own navigators.

***Training:***

- Identify learner needs (pre-assessment) of the volunteers in order to make the training.
- Organize the volunteer training to teach the volunteers how to be navigators.
- Identify own learning needs and take responsibility for seeking out training

***Engage in research activities:***

- Country trainers might be asked to fill out structured diaries, questionnaires, and attendance lists before and after training and coaching/mentoring sessions.
- Country trainers might be asked to engage in face-to-face or group interviews during and after Nav-CARE implementation.

**Part III: Person Specification**

|  | **ESSENTIAL** | **DESIRABLE** |
| --- | --- | --- |
| **Education and Qualifications** | Social or healthcare degree (ie, Nursing, Social Work, Psychology, Sociology, Gerontology, Oncology,…)  Knowledge of the health or social care system either by education or by experience | - Experience with the target population (geriatric oncology) - Knowledge of palliative care - Knowledge of social care, social organizations and community care |
| **Experience** | - Experience in a palliative, nursing, social, gerontology, mental health, OR oncology care setting (inpatient or outpatient). - Experience in mentorship/teaching/training | - Experience in working with or as a volunteer/ working with volunteer organizations |
| **Skills, Knowledge and abilities** | - Excellent communication and presentation skills - Computer literacy with knowledge of Windows based applications - Teaching skills - To be able to listen to the motivation of the volunteers and adjusting to training to their intrinsic motivation | - Networking skills |
| **Attitude, Personal Characteristics** | - Ability to work independently in an organized and methodical way – manage time & meet deadlines - Innovative approach & ability to inspire - An ability to work with a diversity of people, with different backgrounds - Negotiation & problem–solving skills - Approachable and adaptable - Ability to undertake training and direct own learning |  |
