# Supplementary material for: Evaluating a Train-the-Trainer program to implement a navigation program for older people with cancer across six European countries, as part of the EU NAVIGATE project: a Kirkpatrick multi-method evaluation
Source: BMC Health Serv Res. 2026 May 21;26:987. doi: 10.1186/s12913-026-14717-6 (PMC13371289; doi:10.1186/s12913-026-14717-6)
Supplement: Supplementary file 3 — Supplementary Material 3: Appendix 3: Topic guide group interview with local trainers. [file 12913_2026_14717_MOESM3_ESM.docx]

| **Day 1** | **Introduction day** |
| --- | --- |
| 9:00        until 17:00 | Introductions / Program /Getting to know each other and each other’s contexts   - Background NavCARE - Overview EU Navigate study - Organizational chart: Roles, responsibilities and learning/follow up activities - Week program: way of working per module Wrap up day 1 – Selfcare exercise |
| **Day 2** | **Train the Trainer Module 1 and 2** |
| 9:00          until 17:00 | Module 1: Volunteer role   - Introductory roleplay and discussion - Schematic overview module - Country-moderated interactive learning activity + follow up discussion - Contextualization by prof. Wendy Duggleby - Discussion on cultural adaptations - Module 2: Adressing Quality of life (Structured as above) - Wrap up day 2 – Selfcare exercise |
| **Day 3** | **Module 3 and 4 + Coördinator role** |
| 9:00        until 17:00 | Module 3: Advocating for Clients and Families (Structured as above)  Module 4: Facilitating Community Connections (Structured as above)   - Coordinator Role - Specificities coördinator role - Coördinator-trainer dyad   Wrap up day 3 – Selfcare exercise |
| **Day 4** | **Module 5 and 6 + Context analysis and cultural adaptations** |
| 9:00        until 17:00 | Module 5: Promoting Active Engagement (Structured as above)  Module 6: Supporting Virtual Navigation (Structured as above)   - Cultural adaptations - Interviews context analysis   Working on country timelines Wrap up day 4 – Selfcare exercise |
| **Day 5** | **Research, pre-assessment and pre-requisites, Wrap up** |


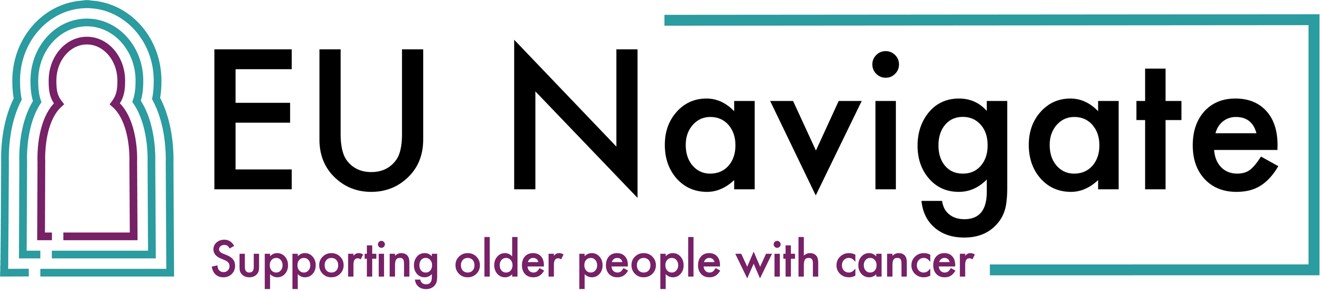
**Overview International Training week April 24^th^ to April 28^th^ 2023**

9:00 Research aspects of intervention (Dr. Lara Pivodic)

- Pre-assessment learner needs
- Background knowledge volunteering in End-of-Life trajectories (CHPCA) until Training Wrap up: Q&A and Evaluations

14:30 Wrap up Lunch
